# Boracic Acid and Its Application

**Published:** 1876-11

**Authors:** Leonard Cane


### ARTICLE VI.

### *Boracic Acid and its Application.*

Dr. Leonard Cane, in the *Lancet*, speaks of the value of this agent as an antiseptic :

The use of boracic acid and the different preparations containing it has been introduced as part of the antiseptic system, and its advantages have been well dwelt upon by Professor Lister, Mr. Godlee and others. But it is as a simple dressing for wounds of all kinds, altogether apart from the antiseptic system, strictly so called, that I wish to draw attention to it.

However great the advantages of Mr. Lister's method of dressing wounds, it is undoubtedly felt by the great majority of surgeons, especially those engaged in private practice, and whose time is often limited, that the details and the time required for their proper performance practically prevent its use in all ordinary cases. In hospital practice, where skilled assistance is always at hand, and in the higher class of purely surgical practice, I believe that the antiseptic mode of dressing wounds is by far the best. But we want something which, while it has to a certain extent the merits, yet is without those tedious details, and which can readily be performed by anyone without assistance.

The preparations of boracic acid have now been rather extensively tried by me for some months, and in all the cases in which they have been used the results have been good, and decidedly better than under the ordinary methods of dressing. The most convenient forms for use are the boracic (boric) lint and cotton-wool, a concentrated watery solution of the acid, and boracic ointment. Boracic lint is prepared by soaking lint in a saturated boiling solution of the acid. On drying the lint a copious deposit of fine flaky crystals takes place between its fibres. Cotton-wool may be similarly served, and when dried and carefully picked out forms a very useful dressing. The concentrated solution is made by dissolving the acid in boiling water to saturation. The ointment is made by rubbing down one drachm of the acid with one ounce of simple ointment, or benzoated lard.

Boracic acid, unlike most antiseptic agents, is bland and unirritating; and, whilst its non volatility renders it less useful in some cases than carbolic acid, its great superiority to this and to chloride of zinc resides in its unirritating nature. The boracic lint is best used as a dry dressing; and for recent wounds, where simplicity is desired, it has no equal. A pad of lint applied immediately over the wound, and kept in place by pieces of strapping, is all that is required, and union by first intention is a common result.

For boils on the neck and elsewhere the boracic lint is an excellent application; a piece large enough to hide the boil, and covered with a piece of gutta-percha tissue, often gives great relief. For carbuncles and other cases, in which it is desired to apply a poultice, I have found the new "instantaneous poultice," prepared from iceland moss by Messrs. Rigollot, a capital and efficient remedy. The poultice should be prepared by soaking it for a short time in the boracic lotion, and when applied should be covered with gutta percha tissue.

Lastly, in some of the vegetable parasitic diseases, such as pityriais ver sicolor, tinea circinata, etc., the boracic lotion and ointment will often be found serviceable.

Briefly to sum up the advantages of boracic acid:—

1. It is an antiseptic which does not irritate and inflame and so allows the natural processes of healing to go on without much interruption.

2. It is exceedingly simple in its application, and can be used apart from all the details required by a thoroughly antiseptic method.

3. It can be used in the shape of the lint lotion, cotton-wool, etc., in combination with most other methods of treatment.

4. Its cost is trifling; and though this is of secondary importance, it is a feature of the treatment which will recommend its employment in work-house infirmaries and in dispensary and parish practice.

---

